# Supplementary material for: Modeling glioblastoma heterogeneity as a dynamic network of cell states
Source: Mol Syst Biol. 2021 Sep 16;17(9):e10105. doi: 10.15252/msb.202010105 (PMC8444284; doi:10.15252/msb.202010105)
Supplement: Supplementary file 6 — Source Data for Figure 5 [file MSB-17-e10105-s004.zip › Figure5A_sourcedata/GSEA_3017/nosigdb_stateC.GseaPreranked.1622795002785/gsea_report_for_na_pos_1622795002785.html]

Report for na\_pos 1622795002785 [GSEA]

| GS  follow link to MSigDB | GS DETAILS | SIZE | ES | NES | NOM p-val | FDR q-val | FWER p-val | RANK AT MAX | LEADING EDGE || 1 | RICHARDS\_NOWAKOWSKI\_SCIENCE\_2017\_MGE\_DIV\_UPREG | Details ... | 123 | 0.80 | 7.00 | 0.000 | 0.000 | 0.000 | 194 | tags=96%, list=32%, signal=113% |
| 2 | RICHARDS\_NOWAKOWSKI\_SCIENCE\_2017\_IPC\_DIV2\_UPREG | Details ... | 135 | 0.75 | 6.85 | 0.000 | 0.000 | 0.000 | 194 | tags=92%, list=32%, signal=105% |
| 3 | RICHARDS\_NOWAKOWSKI\_SCIENCE\_2017\_MGE\_IPC2\_UPREG | Details ... | 109 | 0.79 | 6.84 | 0.000 | 0.000 | 0.000 | 194 | tags=96%, list=32%, signal=117% |
| 4 | DOROSZKO\_U3180MG\_CLUSTER\_3 | Details ... | 100 | 0.79 | 6.77 | 0.000 | 0.000 | 0.000 | 191 | tags=97%, list=32%, signal=119% |
| 5 | DOROSZKO\_U3054MG\_CLUSTER\_4 | Details ... | 108 | 0.78 | 6.65 | 0.000 | 0.000 | 0.000 | 191 | tags=96%, list=32%, signal=116% |
| 6 | GAROFANO\_IAVARONE\_PPR\_SUBTYPE | Details ... | 77 | 0.70 | 5.71 | 0.000 | 0.000 | 0.000 | 192 | tags=94%, list=32%, signal=120% |
| 7 | RICHARDS\_NOWAKOWSKI\_SCIENCE\_2017\_IPC\_DIV1\_UPREG | Details ... | 101 | 0.65 | 5.34 | 0.000 | 0.000 | 0.000 | 175 | tags=80%, list=29%, signal=94% |
| 8 | LARSSON\_STATE\_4 | Details ... | 50 | 0.75 | 5.12 | 0.000 | 0.000 | 0.000 | 151 | tags=92%, list=25%, signal=113% |
| 9 | DOROSZKO\_U3013MG\_CLUSTER\_2 | Details ... | 49 | 0.72 | 5.09 | 0.000 | 0.000 | 0.000 | 174 | tags=96%, list=29%, signal=124% |
| 10 | RICHARDS\_NOWAKOWSKI\_SCIENCE\_2017\_MGE\_IPC1\_UPREG | Details ... | 76 | 0.57 | 4.40 | 0.000 | 0.000 | 0.000 | 146 | tags=70%, list=24%, signal=81% |
| 11 | RICHARDS\_NOWAKOWSKI\_SCIENCE\_2017\_MGE\_RG2\_UPREG | Details ... | 96 | 0.53 | 4.30 | 0.000 | 0.000 | 0.000 | 179 | tags=72%, list=30%, signal=86% |
| 12 | NB\_KI\_CLUSTER5 | Details ... | 32 | 0.72 | 4.14 | 0.000 | 0.000 | 0.000 | 191 | tags=100%, list=32%, signal=139% |
| 13 | SEURAT\_CELLCYCLE | Details ... | 28 | 0.74 | 4.07 | 0.000 | 0.000 | 0.000 | 106 | tags=82%, list=18%, signal=95% |
| 14 | FELDMAN\_2020\_G1 | Details ... | 54 | 0.57 | 3.96 | 0.000 | 0.000 | 0.000 | 142 | tags=76%, list=24%, signal=91% |
| 15 | FELDMAN\_2020\_S-G2 | Details ... | 95 | 0.49 | 3.95 | 0.000 | 0.000 | 0.000 | 177 | tags=73%, list=30%, signal=87% |
| 16 | RICHARDS\_NOWAKOWSKI\_SCIENCE\_2017\_MGE\_IPC3\_UPREG | Details ... | 31 | 0.66 | 3.90 | 0.000 | 0.000 | 0.000 | 171 | tags=90%, list=29%, signal=120% |
| 17 | RICHARDS\_ZHONG\_NATURE\_2018\_NPCS\_UPREG | Details ... | 46 | 0.59 | 3.88 | 0.000 | 0.000 | 0.000 | 139 | tags=74%, list=23%, signal=89% |
| 18 | FELDMAN\_2020\_S | Details ... | 66 | 0.49 | 3.65 | 0.000 | 0.000 | 0.000 | 191 | tags=76%, list=32%, signal=99% |
| 19 | SUVA\_G1S\_PMID\_31327527 | Details ... | 16 | 0.74 | 3.28 | 0.000 | 0.000 | 0.000 | 143 | tags=94%, list=24%, signal=120% |
| 20 | RICHARDS\_NEFTEL\_CELL\_2019\_G1\_S | Details ... | 16 | 0.74 | 3.17 | 0.000 | 0.000 | 0.000 | 143 | tags=94%, list=24%, signal=120% |
| 21 | RICHARDS\_NOWAKOWSKI\_SCIENCE\_2017\_RG\_DIV1\_UPREG |  | 197 | 0.32 | 3.07 | 0.000 | 0.000 | 0.000 | 194 | tags=55%, list=32%, signal=54% |
| 22 | RICHARDS\_NOWAKOWSKI\_SCIENCE\_2017\_NIN2\_UPREG |  | 22 | 0.59 | 2.96 | 0.000 | 0.000 | 0.000 | 196 | tags=86%, list=33%, signal=124% |
| 23 | RICHARDS\_NOWAKOWSKI\_SCIENCE\_2017\_NIN1\_UPREG |  | 22 | 0.57 | 2.87 | 0.000 | 0.000 | 0.000 | 166 | tags=77%, list=28%, signal=103% |
| 24 | VANGRONINGEN\_ADRN |  | 39 | 0.47 | 2.87 | 0.000 | 0.000 | 0.000 | 177 | tags=72%, list=30%, signal=95% |
| 25 | RICHARDS\_NOWAKOWSKI\_SCIENCE\_2017\_RG\_EARLY\_UPREG |  | 79 | 0.36 | 2.84 | 0.000 | 0.000 | 0.000 | 139 | tags=48%, list=23%, signal=54% |
| 26 | RICHARDS\_NEFTEL\_CELL\_2019\_NPC2 |  | 16 | 0.62 | 2.72 | 0.000 | 0.000 | 0.000 | 152 | tags=81%, list=25%, signal=106% |
| 27 | SUVA\_NPC2\_PMID\_31327527 |  | 16 | 0.62 | 2.71 | 0.000 | 0.000 | 0.000 | 152 | tags=81%, list=25%, signal=106% |
| 28 | SUVA\_NPC1\_PMID\_31327527 |  | 17 | 0.59 | 2.70 | 0.000 | 0.000 | 0.000 | 166 | tags=82%, list=28%, signal=111% |
| 29 | RICHARDS\_NEFTEL\_CELL\_2019\_NPC1 |  | 17 | 0.59 | 2.67 | 0.000 | 0.000 | 0.000 | 166 | tags=82%, list=28%, signal=111% |
| 30 | RICHARDS\_NOWAKOWSKI\_SCIENCE\_2017\_RG\_DIV2\_UPREG |  | 199 | 0.27 | 2.59 | 0.000 | 0.000 | 0.002 | 191 | tags=51%, list=32%, signal=50% |
| 31 | SINGH\_SOX2\_UPREGULATED\_TRANSCRIPTS\_PMID\_28122245 |  | 53 | 0.36 | 2.52 | 0.000 | 0.000 | 0.004 | 96 | tags=43%, list=16%, signal=47% |
| 32 | GAROFANO\_IAVARONE\_LIST\_OF\_NEURAL\_PROGENITOR\_MARKER\_GENES |  | 27 | 0.46 | 2.47 | 0.000 | 0.000 | 0.004 | 145 | tags=70%, list=24%, signal=89% |
| 33 | IVY\_CT |  | 60 | 0.34 | 2.42 | 0.000 | 0.000 | 0.009 | 145 | tags=53%, list=24%, signal=63% |
| 34 | TF\_CENSUS\_TAIPALE |  | 65 | 0.31 | 2.34 | 0.000 | 0.000 | 0.011 | 117 | tags=40%, list=20%, signal=44% |
| 35 | RICHARDS\_NOWAKOWSKI\_SCIENCE\_2017\_NIN5\_UPREG |  | 21 | 0.47 | 2.34 | 0.000 | 0.000 | 0.011 | 110 | tags=57%, list=18%, signal=68% |
| 36 | RICHARDS\_NOWAKOWSKI\_SCIENCE\_2017\_IN\_CTX\_MGE1\_UPREG |  | 22 | 0.46 | 2.32 | 0.003 | 0.000 | 0.012 | 97 | tags=50%, list=16%, signal=58% |
| 37 | RICHARDS\_NOWAKOWSKI\_SCIENCE\_2017\_NIN3\_UPREG |  | 21 | 0.46 | 2.30 | 0.000 | 0.000 | 0.015 | 95 | tags=48%, list=16%, signal=55% |
| 38 | RICHARDS\_NOWAKOWSKI\_SCIENCE\_2017\_IPC\_NEN1\_UPREG\_1 |  | 19 | 0.46 | 2.18 | 0.000 | 0.001 | 0.038 | 258 | tags=89%, list=43%, signal=153% |
| 39 | RICHARDS\_VERHAAK\_CANCERCELL\_2010\_PRONEURAL |  | 22 | 0.40 | 2.03 | 0.005 | 0.004 | 0.119 | 102 | tags=50%, list=17%, signal=58% |
| 40 | DALMO\_BARCODENUMBER\_UNPUB |  | 41 | 0.32 | 2.00 | 0.000 | 0.005 | 0.138 | 137 | tags=51%, list=23%, signal=62% |
| 41 | RICHARDS\_NOWAKOWSKI\_SCIENCE\_2017\_IN\_CTX\_CGE1\_UPREG |  | 20 | 0.41 | 1.99 | 0.005 | 0.005 | 0.154 | 186 | tags=65%, list=31%, signal=91% |
| 42 | RICHARDS\_NOWAKOWSKI\_SCIENCE\_2017\_U2\_UPREG |  | 197 | 0.18 | 1.84 | 0.000 | 0.012 | 0.330 | 139 | tags=34%, list=23%, signal=30% |
| 43 | OLSEN\_ADRENERGIC |  | 18 | 0.38 | 1.76 | 0.013 | 0.019 | 0.490 | 135 | tags=67%, list=23%, signal=84% |
| 44 | RICHARDS\_NOWAKOWSKI\_SCIENCE\_2017\_IPC\_NEN1\_UPREG |  | 36 | 0.29 | 1.72 | 0.021 | 0.024 | 0.573 | 190 | tags=56%, list=32%, signal=77% |
| 45 | RICHARDS\_CAHOY\_JNEUROSCI\_2010\_ASTRO\_YOUNG |  | 118 | 0.19 | 1.66 | 0.005 | 0.032 | 0.676 | 158 | tags=42%, list=26%, signal=45% |
| 46 | RICHARDS\_NOWAKOWSKI\_SCIENCE\_2017\_NIN4\_UPREG |  | 20 | 0.29 | 1.44 | 0.066 | 0.095 | 0.968 | 166 | tags=50%, list=28%, signal=67% |
| 47 | RICHARDS\_NOWAKOWSKI\_SCIENCE\_2017\_IPC\_NEN3\_UPREG |  | 32 | 0.24 | 1.39 | 0.111 | 0.116 | 0.986 | 16 | tags=16%, list=3%, signal=15% |
| 48 | RICHARDS\_NOWAKOWSKI\_SCIENCE\_2017\_IN\_CTX\_CGE2\_UPREG |  | 27 | 0.24 | 1.34 | 0.141 | 0.147 | 0.996 | 97 | tags=33%, list=16%, signal=38% |
| 49 | GAROFANO\_IAVARONE\_NEU\_SUBTYPE |  | 16 | 0.31 | 1.33 | 0.154 | 0.146 | 0.997 | 105 | tags=38%, list=18%, signal=44% |
| 50 | KRONA\_ROSEN\_WHITE\_MATTER\_INVASION |  | 26 | 0.24 | 1.25 | 0.206 | 0.204 | 0.998 | 93 | tags=31%, list=16%, signal=35% |
| 51 | RICHARDS\_NOWAKOWSKI\_SCIENCE\_2017\_NEN\_EARLY2\_UPREG |  | 38 | 0.18 | 1.11 | 0.324 | 0.351 | 1.000 | 192 | tags=47%, list=32%, signal=65% |
| 52 | RICHARDS\_NOWAKOWSKI\_SCIENCE\_2017\_U3\_UPREG |  | 46 | 0.15 | 1.03 | 0.418 | 0.450 | 1.000 | 171 | tags=43%, list=29%, signal=56% |
| 53 | RICHARDS\_NOWAKOWSKI\_SCIENCE\_2017\_NEN\_LATE\_UPREG |  | 21 | 0.21 | 1.02 | 0.461 | 0.450 | 1.000 | 42 | tags=19%, list=7%, signal=20% |
| 54 | NB\_KI\_CLUSTER2 |  | 25 | 0.18 | 0.99 | 0.456 | 0.490 | 1.000 | 192 | tags=52%, list=32%, signal=73% |
| 55 | ALMSTEDT\_11Q\_MSIGDB |  | 23 | 0.19 | 0.96 | 0.474 | 0.518 | 1.000 | 115 | tags=35%, list=19%, signal=41% |
| 56 | RICHARDS\_NOWAKOWSKI\_SCIENCE\_2017\_EN\_V1\_1\_UPREG |  | 67 | 0.07 | 0.57 | 0.975 | 0.957 | 1.000 | 454 | tags=88%, list=76%, signal=326% |
Table: Gene sets enriched in phenotype **na **[plain text format]****
